# Biomechanical Responses of the Brain in Swine Subject to Free-Field Blasts

**DOI:** 10.3389/fneur.2016.00179

**Published:** 2016-10-24

**Authors:** Ke Feng, Liying Zhang, Xin Jin, Chaoyang Chen, Srinivasu Kallakuri, Tal Saif, John Cavanaugh, Albert King

**Affiliations:** ^1^Department of Biomedical Engineering, Wayne State University, Detroit, MI, USA

**Keywords:** free-field blast, head kinematics, traumatic brain injury, swine, intracranial pressure

## Abstract

Blast-induced traumatic brain injury (bTBI) is a signature wound of modern warfare. The current incomplete understanding of its injury mechanism impedes the development of strategies for effective protection of bTBI. Despite a considerable amount of experimental animal studies focused on the evaluation of brain neurotrauma caused by blast exposure, there is very limited knowledge on the biomechanical responses of the gyrenecephalic brain subjected to primary free-field blast waves imposed *in vivo*. This study aims to evaluate the external and internal mechanical responses of the brain against different levels of blast loading with Yucatan swine in free field. The incident overpressure (IOP) was generated using 3.6 kg of C4 charge placed at three standoff distances from the swine. Five swine were exposed to a total of 19 blasts. The three average peak IOP pressure levels in this study were 148.8, 278.9, and 409.2 kPa as measured by a pencil probe. The duration of the first positive wave was in the range of 2.1–3 ms. Pressure changes in the brain and head kinematics were recorded with intracranial pressure (ICP) sensors, linear accelerometers, and angular rate sensors. The corresponding average peak ICPs were in the range of 79–143, 210–281, and 311–414 kPa designated as low, medium, and high blast level, respectively. Peak head linear accelerations were in the range of 120–412 g. A positive correlation between IOP and its corresponding biomechanical responses of the brain was also observed. These experimental data can be used to validate computer models of bTBI.

## Introduction

Exposure to blasts from improvised explosive devices has become the most common cause of injury to our soldiers (1). The causes of blast-induced injury are usually complicated and can be due to one or more mechanisms (2). Unlike the other well-studied categories of injury, the mechanisms and injury criteria of primary blast injury that directly result from the transmission of shock waves to the body are the least well known although the physics of blast waves are well characterized (3, 4). In the past 70 years, researchers have performed substantial work in this field and have been able to define the biological tolerance levels of the most vulnerable air-filled organs to primary blast, such as the ears and lungs (5–7). These criteria were developed based on the peak overpressure and duration. With the improved design and extensive use of personal protective equipment, mortality rate, and severity of injuries level from blast explosives have significantly decreased in recent years (8). However, there is an increase in mild to moderate closed-head traumatic brain injury (TBI) due to blasts (9). Patients with this type of injury are often found without any physically visible defects or brain damage identifiable by imaging, but show persistent symptoms such as fatigue, headaches, and delayed recall of memory (10–12). It is still not clear how a blast wave interacts with the head and transfers energy through various parts of the cranium to cause brain injury (13, 14). In order to develop better head protection from blast, the injury mechanisms and injury thresholds related to primary blast-induced traumatic brain injury (bTBI) need to be defined based on the characteristic parameters of the blast wave, mechanical response of the brain, and the skull as well as injury to the brain tissue.

Most studies have utilized shock tubes to produce overpressure (15). The distinct advantages of shock tube are its economical function for repeated tests in laboratories and repeatability of the desired overpressure by using membranes of the same thickness. Its ease of scheduling compared with field tests, including severe restrictions of weather, explosives handling, and availability of personnel, make the shock tube tests a prevalent choice. Additionally, shock tube tests have the ability to apply more advanced instrumentation and diagnostics than feasible at field trials. However, the overpressure generated in conventional shock tubes is different from a free-field blast wave. Some shock tubes generate shockwaves with prolonged positive duration outside the realm of real world situations (16, 17). The test animal size is limited by the shock tube test section and there could be non-negligible complex reflections within the shock tube. These characteristics would make the corresponding mechanical responses different from those in free-field blasts, in which test subjects can be exposed to a simple Friedlander wave without interference from reflections. Additionally, blast testing in the open field with proper settings can provide relevant physical parameters of blast conditions similar to those in the battlefield. In the real world, reflections of the shock wave from the ground are inevitable. Due to the complexities of gas-dynamic shock reflection phenomena, the reflected and incident waves merge into a new wave front called the “Mach stem” (18). To minimize the effect of reflected waves, it is necessary to locate the “triple point” within the “Mach stem” region utilizing appropriate standoff distances and the heights of burst.

The measurement of shock wave propagation patterns in an *in vivo* brain remains a significant challenge. Much research has been conducted with rats and swine to measure the intracranial pressure (ICP) responses in the brain using shock tubes (19–23). However, in most of the studies, only a few sensors were installed (19, 20, 22). In some cases, there was no detailed description of sensor locations (19, 22). This lack of accurate information constitutes an impediment to a full understanding of how a pressure wave interacts with various parts of the brain. In addition, the brain structures and skull thickness vary widely between different animals. Yucatan swine, 6–8 months in age, have a similar body mass (50–60) and skull thickness (6–17 mm) as human. Biomechanical responses of swine to blast overpressure are expected to be closer to those of the human and, thus, it would be more appropriate to study them instead of the small animals like rodents.

Additionally, computational modeling can help elucidate the comprehensive responses of the head and brain to blast (24–27). One of the hypotheses is that the scalp may exacerbate the pressure effects in the brain (24). Others have shown that the skull flexure due to blast is a potential mechanism (25). The distribution of ICP and the kinetics of the head have been simulated in several models (26–28). However, experimental data are still needed to validate these models.

In view of the limitations in bTBI research, there is a need to develop reliable and more operationally relevant animal models. To characterize the effects of free-field blast on the head, this study exposed swine to free-field blasts generated by explosives at different incident overpressure (IOP) levels. Thus, the aim of this study was to provide data on the mechanical responses of the swine in primary bTBI. To the best of our knowledge, this would be the first set of published experimental biomechanical data from swine subjected to free-field blast overpressure.

## Materials and Methods

### Animal Preparation

**Figure 1:**
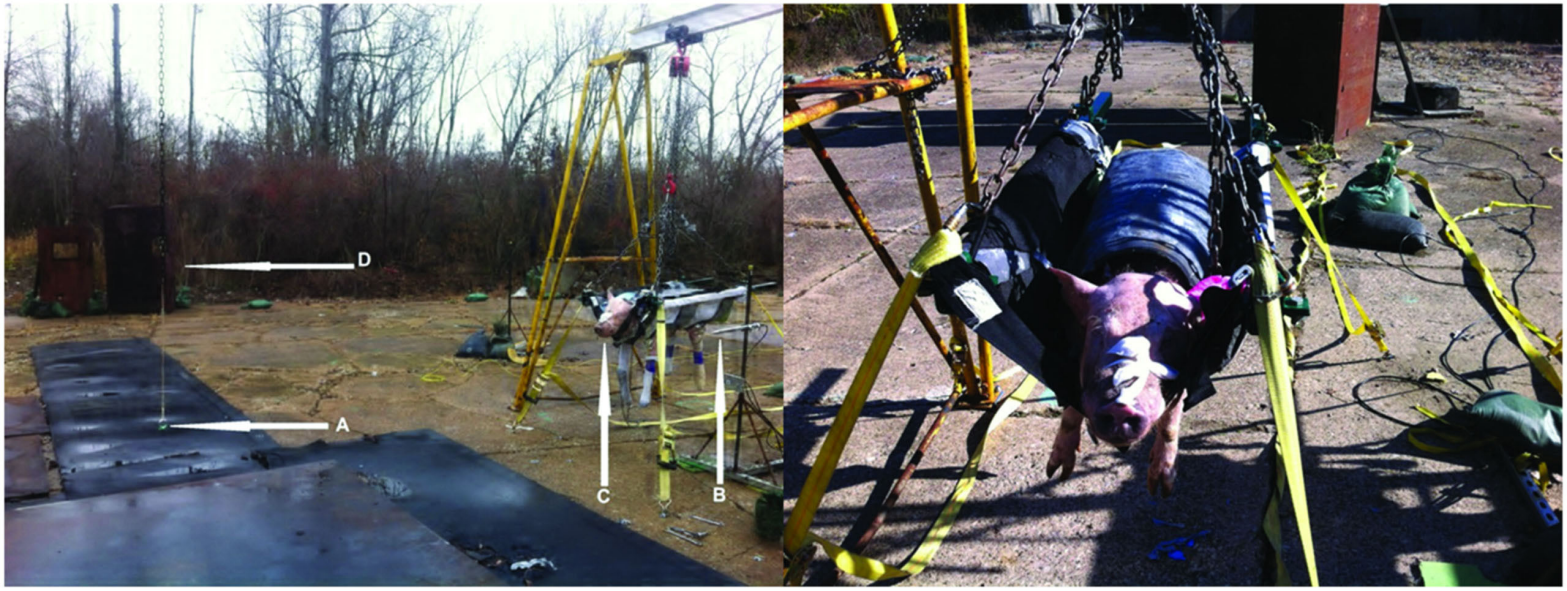
**Blast experiment test set-up**. The photograph on the left shows the anesthetized swine placed in a lead-covered sling hanging from a metal beam that is attached to two A-Frames. The arrow at “A” points to the C4 charge. Arrow B shows the pencil pressure sensor used to record the IOP. Arrow C shows the head of the test animal facing in the direction of the oncoming blast wave front. Arrow D shows the location of high-speed cameras and their shielding. The right photograph shows the swine in a sling anchored to the A-frames and covered with a foam-lined lead shield protecting the torso.

The research protocol for this study was reviewed and approved by the Institutional Animal Care and Use Committee and the USAMRMC Animal Care and Use Research Office (ACURO). Five instrumented Yucatan swine (age 6–8 months, weight 50–60 kg) were exposed to repeated frontal free-field blasts to collect biomechanical data. Before instrumentation, all swine were acclimated for 6–8 days before testing to their new housing conditions. On the test date, the animal was transported ~145 km (90 miles) in an ambulance to the blast site (ARES, Port Clinton, OH, USA) under anesthesia (ketamine 20 mg/kg intramuscular and xylazine 2 mg/kg intramuscular). The ambulance was equipped with an examination table and equipment for physiological monitoring to ensure maintenance of the proper anesthetic level. Once at the test site, a surgical procedure to install ICP sensors was performed. Blood pressure, oxygen saturation, heart rate, and respiratory rate were monitored before and in between blast exposures. During the tests, the animal was maintained under anesthesia (propofol 12–20 mg/kg/hr constant-rate infusion). To expose the swine to open field blast, the animal was placed prone in a specially designed canvas sling with holes for the extremities. The sling was supported by a steel body frame that was suspended from a metal I-beam that was 3.7 m off the ground. The I-beam was supported by two steel A-frames, as shown in Figure 1. The body frame was tied down to the A-frames with straps to prevent excessive motion due to the blast wind. To prevent thoracic injuries from primary blast, the torso was wrapped in a lead sheet that had a density of 39 kg/m^2^. A piece of 0.3175-cm-thick foam padding was placed between of the lead sheet and the animal. The snout of the animals was secured by webbing material to support the head during blast tests.

### Free-Field Blast Procedure

C4, weighing 3.6 kg, was packed into a sphere and detonated to generate the blast waves. The height of burst (HOB) was controlled by suspending the C4 from a metal chain and a 5-cm-thick metal plate was placed under the charge to eliminate debris and assure consistency of the reflected wave. To generate different levels of IOP, the explosive was placed at varying distances from the pig’s head. Three levels of IOP were used in this study. Since the goal was to evaluate mechanical responses due to non-fatal primary bTBI, the pressure levels were selected based on previous swine studies that were tested using shock tubes (19, 23). These three pre-determined peak IOP levels were nominally designated as low (150 kPa), medium (300 kPa), and high (400 kPa). To attain the best approximation of an ideal Friedlander waveform, the height of the triple point as a function of the horizontal distance from a given charge weight was calculated for a range of HOB (29, 30). The HOB of the charge was computed to be 0.8–0.91 m with the height of the head of the test subject at or less than 0.91 m. The estimated horizontal distances from the charge and the HOB to produce the three different blast pressure levels were further verified by a finite element simulation (ConWep card in LS-Dyna, LSTC, Livermore, CA, USA).

To record the IOP profile during each test, a pencil pressure sensor (137B24B, PCB Piezotronics, Depew, NY, USA) was placed near the animal at the level of its eyes while two backup pencil sensors were placed at the same height along a circular arc with a radius equal to the desired standoff distance. Each sensor was mounted on a metal frame that was bolted to the concrete ground. A total of nine blast tests run at three standoff distances were conducted to validate and finalize the calculated standoff distances based on the IOP measured from pencil probes. Then for each animal, a series of nine blast tests were conducted at three pressure levels and in three orthogonal orientations – frontal, lateral, and rear. The current communication reports the results from frontal blast tests only. Lateral and rear blasts results will be presented separately.

### Instrumentation and Data Acquisition

**Figure 2:**
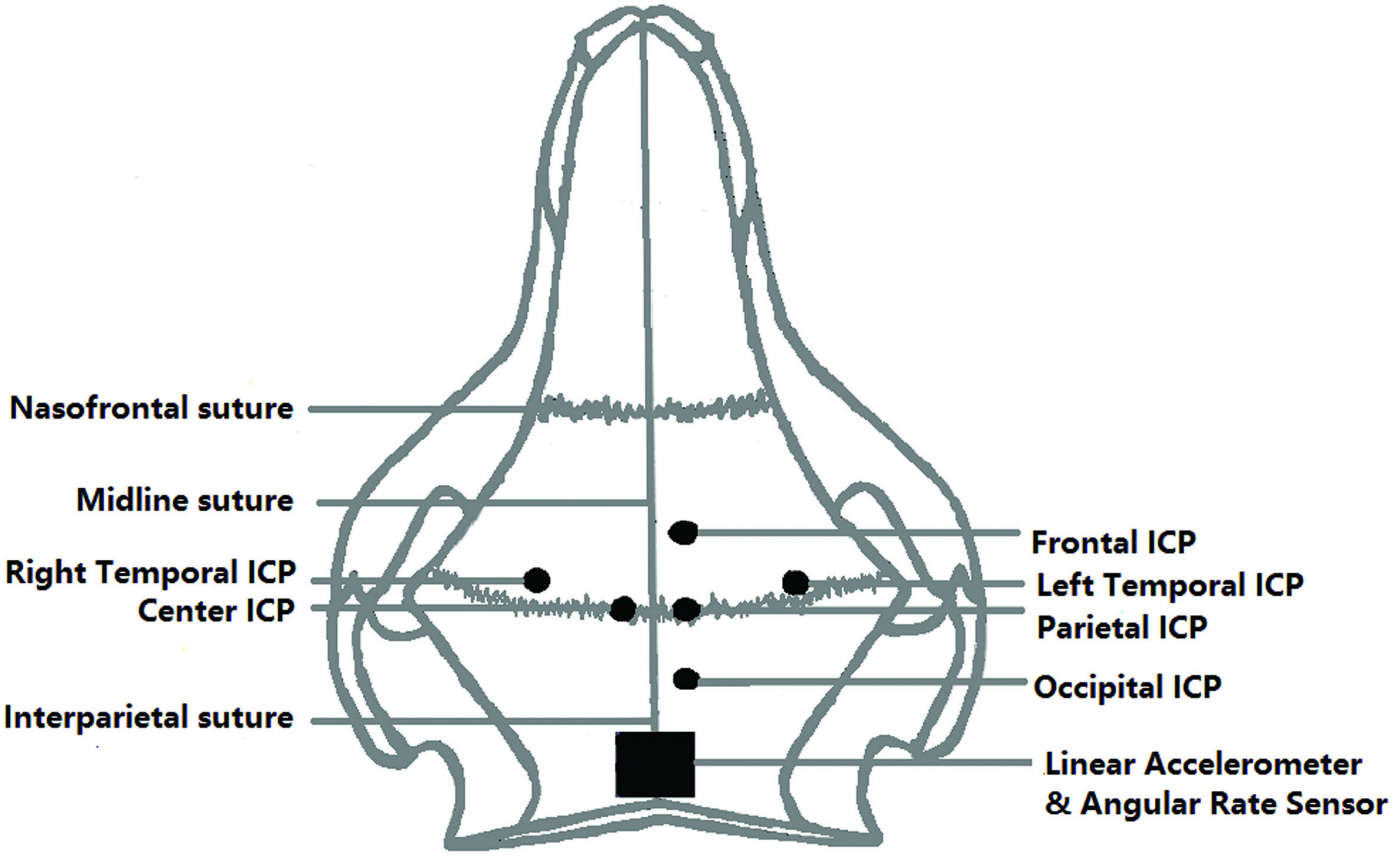
**Top view of the location of ICP sensors relative to the skull of the swine**.

Intracranial pressure transducers (XCL-072-100A, Kulite, CA, USA) were installed in the frontal, occipital, left and right temporal and parietal lobe, and at the center of the brain. Holes in the skull were drilled using a drill bit with a stop collar and the transducers were installed and secured with a 1/4-inch diameter threaded copper hollow fitting, equipped with a threaded cap (Dorman, Colmar, PA, USA). A 1/8-inch diameter cannula was inserted into the brain through the copper fitting to guide the pressure sensor into place and then withdrawn. A customized rubber cap was applied to seal any possible gap between the cable and the cannula. The vertical distance between the brain surface and the tip of frontal, parietal, temporal, and occipital ICP transducers was 5–7 mm. The depth of the center ICP transducer was 10–12 mm. The diameter of the pressure transducers was 1.9 mm. The three linear accelerometers (7264D-2KTZ-2-360, Meggitt’s Endevco, CA, USA) and the three angular rate sensors (ARS-50K-HG, DTS, CA, USA) were fastened to a single aluminum block (ARS HG Triax block, DTS, CA, USA) and installed on top of the skull to monitor the motion of the head. To ensure rigid attachment of the accelerometer block, a 4 cm × 4 cm of scalp was removed from the skull posterior to the lambda. Its location is shown in Figure 2, which also shows the approximate locations of the six ICP transducers. The *X*-axis was defined as the axial direction of the blast, the *Y*-axis was defined as being perpendicular to the sagittal plane of swine’s head, and the *Z*-axis was normal to the transverse surface of the swine’s head at the location of the sensor block. The detailed sensor locations are presented in Table 1. After all the tests were done, the instrumented animal was euthanized at the blast site with an overdose of sodium pentobarbital (120 mg/kg, intraperitoneally). A parallel group of non-instrumented animals also underwent similar blast tests for histological and biomarker studies. Results of these studies are reported separately.

**Table 1:** **A summary of the location of all sensors**.

Sensor	Location
Frontal ICP	2.5 cm anterior to Bregma, 0.5 cm left of midline
Parietal ICP	At the level of Bregma, 0.5 cm left of midline
Center ICP	At the level of Bregma, 0.5 cm right of midline
Left temporal ICP	0.5 cm anterior to Bregma, 1.0 cm left of midline
Right temporal ICP	0.5 cm anterior to Bregma, 1.0 cm right of midline
Occipital ICP	1.5 cm posterior to Bregma, 0.5 cm left of midline
Accelerometer block	2.5 cm posterior to Bregma, on the midline

The IOP and the biomechanical responses of the head, including the ICP, head linear acceleration, and head angular velocity were acquired at a sampling rate of 1 MHz using the DeweSoft (SIRUS, Dewe Soft LLC., OH, USA) and DEWETRON data acquisition system (Dewe-3020, DEWETRON Inc. RI, USA). Two high-speed digital camera systems (GX-8, HX-1, NAC Image, MN, USA) were set up to record high-resolution videos of the blast event. One of the cameras was focused on the head of the instrumented swine and ran at 20,000 frames/second (fps). The other camera ran at 10,000 fps and was designed to obtain an overall view of the blast wave propagation from the C4 charge to the swine test subject. It provided evidence of the uniformity of the blast based on the sphericity of fireball. Data from the sensors acquired from swine were synchronized with video data from both cameras.

### Data Processing and Analysis

Incident overpressure and ICP data were systematically filtered with a 100 and 10 kHz Butterworth low pass filter, respectively. Baseline noise was filtered out but the pressure data retained at least 90% of their original values. Linear acceleration data were filtered with 5 kHz and angular velocity data were filtered with a 2 kHz Butterworth low pass filter. High-frequency content relevant to skull flexure and brain/skull dynamics was filtered in order to correlate results with SAE injury criteria for global head motion relevant to automobile-crash scenarios. All post data processing and statistical analysis were performed using DIAdem 2012 software (National Instruments Corporation, Austin, TX, USA) and IBM SPSS Statistics (Version 22.0. Armonk, NY, USA). All data were grouped into three IOP levels according to the recorded IOP by the pencil probe located next to the head of the swine. The duration of the blast wave was defined as the time the IOP stayed above ambient pressure and was determined using Diadem. IOP and ICP impulse were defined as the area of the positive phase of the IOP or ICP wave and were obtained through integration. Peak ICP values were determined for each blast for statistical analysis. Linear regression models were constructed to predict the relative relationship between ICP readings within groups. ICP box plots were drawn to show the distribution of pressure within each group. Paired *t*-tests were performed between IOP and ICP at each location at the same blast level. One-way analysis of variance (ANOVA) tests were performed to compare the mean peak ICP readings between various locations at the same blast level and the peak ICP readings at the same location in different blast levels. Average Peak ICP values for each test were correlated with their peak IOP values. The rise time of the ICP was defined as the duration of ICP from the ambient pressure to its peak level. Maximum pressure rise rate was calculated by ICP peak values divided by its rise time.

The peak resultant acceleration was calculated based on the data measured by the three accelerometers. Similarly, the resultant angular velocity was calculated from results acquired by the three angular rate sensors. Linear regression models were used to describe the relationship between the peak resultant acceleration, peak resultant angular velocity, and the peak IOP or IOP impulses. A one-way ANOVA test of peak resultant acceleration was performed between low, medium, and high blast levels. No data at the high blast level were collected due to sensor cable failure and signal anomalies. Independent *t*-tests were performed to compare the resultant angular velocities between the low and medium pressure levels.

## Results

### Intracranial Pressure Response

**Figure 3:**
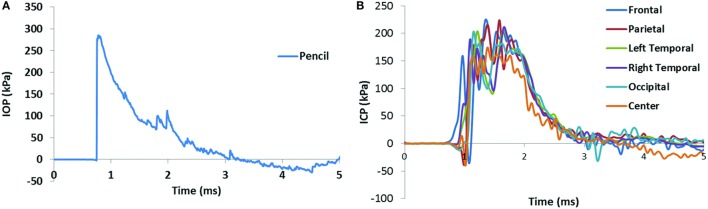
**Pencil reading from a medium level blast (A) and the ICP results in the swine brain from the same blast (B)**. The positive phase duration of the IOP was 2.30 ms, and the impulse of the IOP was 207.7 Pa s.

The results of a total of 19 frontal blasts are reported in this study, using five swine. Plots of a typical set of IOP and ICP curves are shown in Figure 3. The peak IOP, duration, and IOP impulse of each test are summarized in Table 2. In our study, peak IOPs ranged from 143 to 461 kPa. The impulses ranged from 156 to 239 Pa s. The test results were then divided into three pressure level groups based on the IOP results (Table 3). The average peak IOP values were 149, 279, and 409 kPa for the low, medium, and high blast levels, respectively. The average peak ICP at various locations of the brain was in the range of 79–144 kPa at the low blast level, 209–282 kPa at the medium blast level, and 312–415 kPa at the high blast level.

**Table 2:** **A summary of IOPs in this study: their peak value, duration of the first positive wave, and the impulse of the first positive waveform**.

Test ID	Peak incident pressure (kPa)	Duration (ms)	Impulse (Pa s)
1^a^	150.3	2.8	170.3
2^a^	142.7	2.9	155.7
3	150.3	2.9	158.3
4	148.2	2.9	160.3
5	152.4	3.1	161.0
6	218.0	2.1	193.9
7	253.4	2.2	195.7
8	255.2	2.0	194.9
9	324.2	2.3	194.1
10^a^	285.5	2.3	207.7
11^a^	284.1	2.1	198.1
12	285.4	2.0	196.4
13	325.4	2.0	204.0
14	366.0	1.6	205.4
15^a^	441.3	1.7	225.2
16^a^	413.7	1.6	229.6
17^a^	460.6	1.7	239.2
18^a^	341.3	2.4	228.8
19	432.3	2.4	222.9

*^a^Indicates tests in which swine had already expired during testing*.

**Table 3:** **ICP peak values generated by peak IOP at the low, medium, and high levels**.

Test	Biomechanical responses, ICP peak values, mean ± SE						
	IOP	Frontal	Parietal	Left temp	Right temp	Occipital	Center
Low	148.8 ± 1.7	97.6 ± 19.7	144.2 ± 18.0	142.8 ± 0.0	147.9 ± 0.0	78.9 ± 13.4	93.7 ± 17.0
Medium	278.9 ± 13.9	236.5 ± 30.7	276.0 ± 62.4	281.6 ± 35.0	253.1 ± 46.8	209.1 ± 34.5	228.1 ± 29.5
High	409.2 ± 18.9	311.7 ± 29.1	414.6 ± 0.0	386.4 ± 7.1	325.5 ± 8.6	328.2 ± 26.7	327.2 ± 17.0

**Figure 4:**
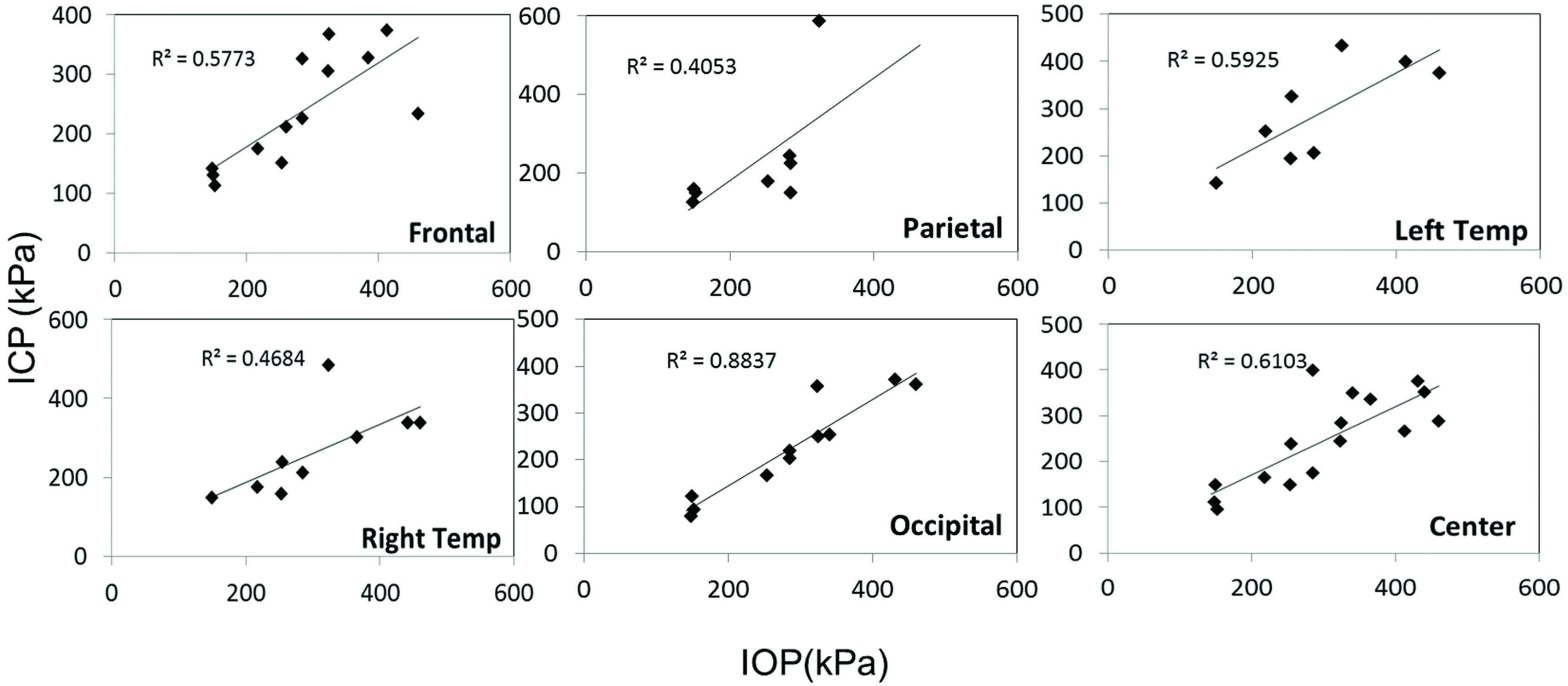
**Scatter plots of ICP vs. IOP at different locations of the brain**. The *x*-axis is the IOP and the *y*-axis is the ICP, both in units of kPa. A linear regression model and *R*^2^ values are shown in each plot.

**Figure 5:**
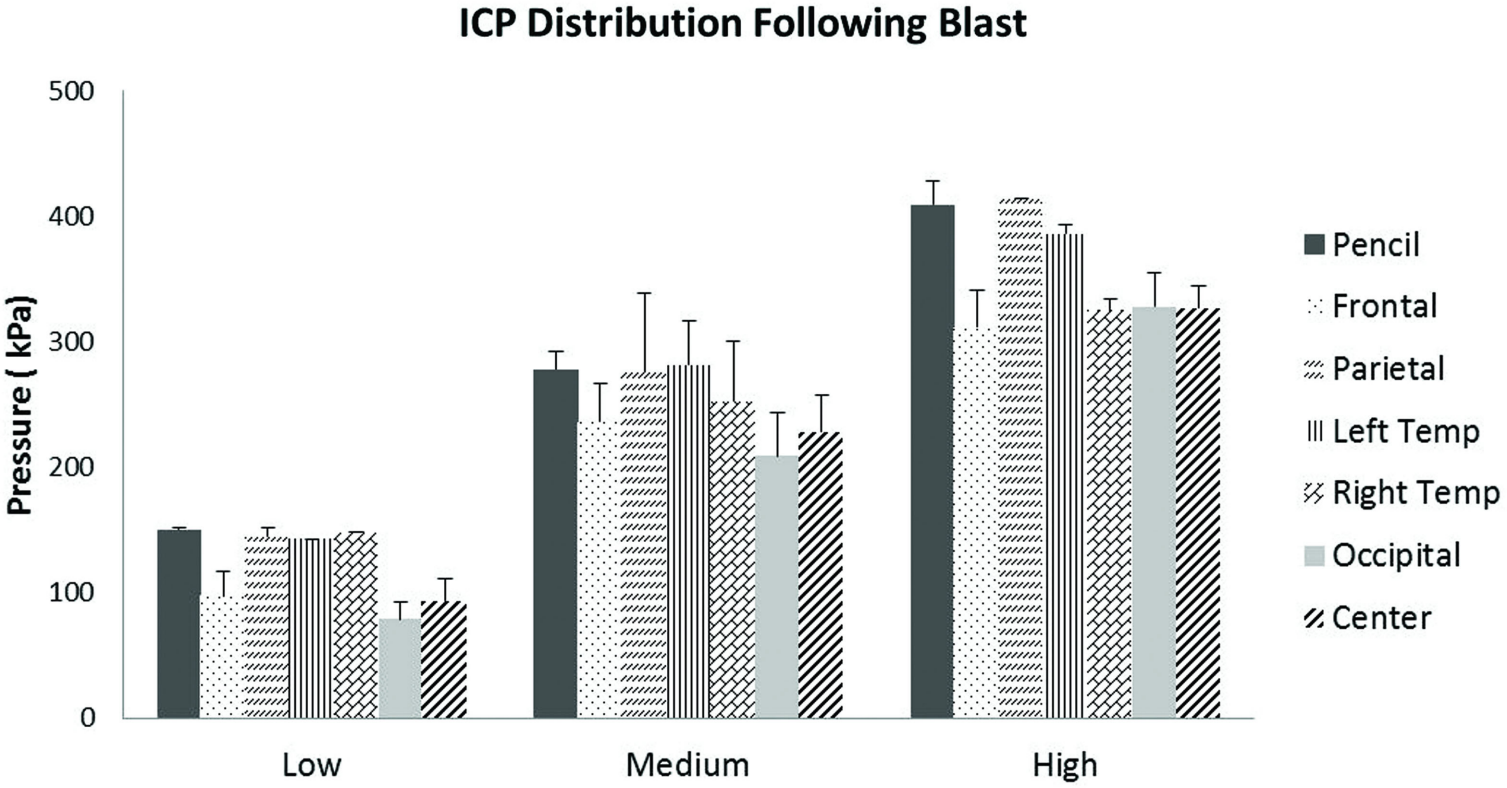
**Peak ICP readings for different levels of blast**. Peak ICPs in different regions of the brain within each blast level group were not statistically different from each other.

**Figure 6:**
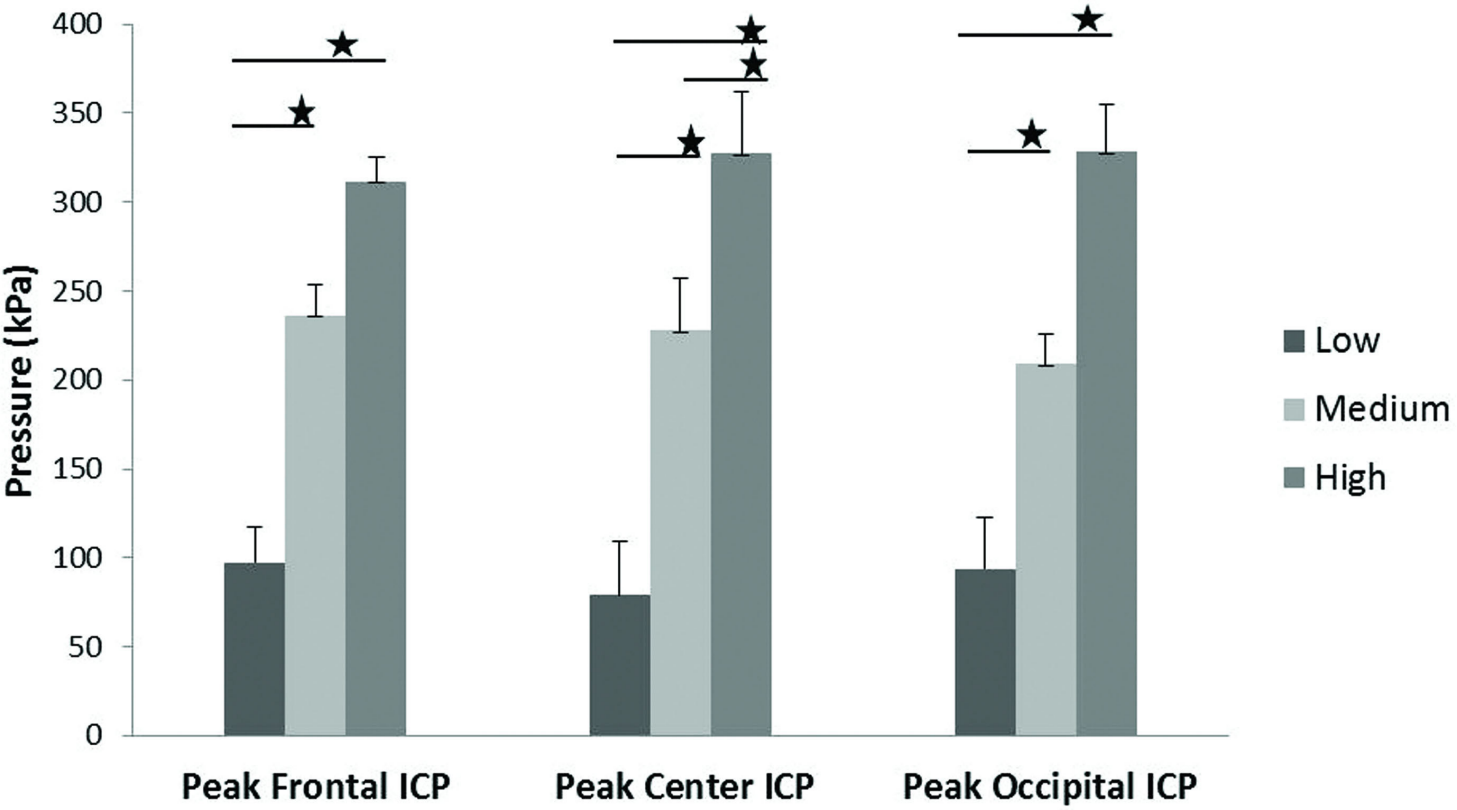
**ICP peak values in the frontal, central, and occipital regions of the brain showed a significant increase with increasing blast levels**. Student’s *t*-tests indicated a significant difference between blast levels (*p < 0.05).

**Figure 7:**
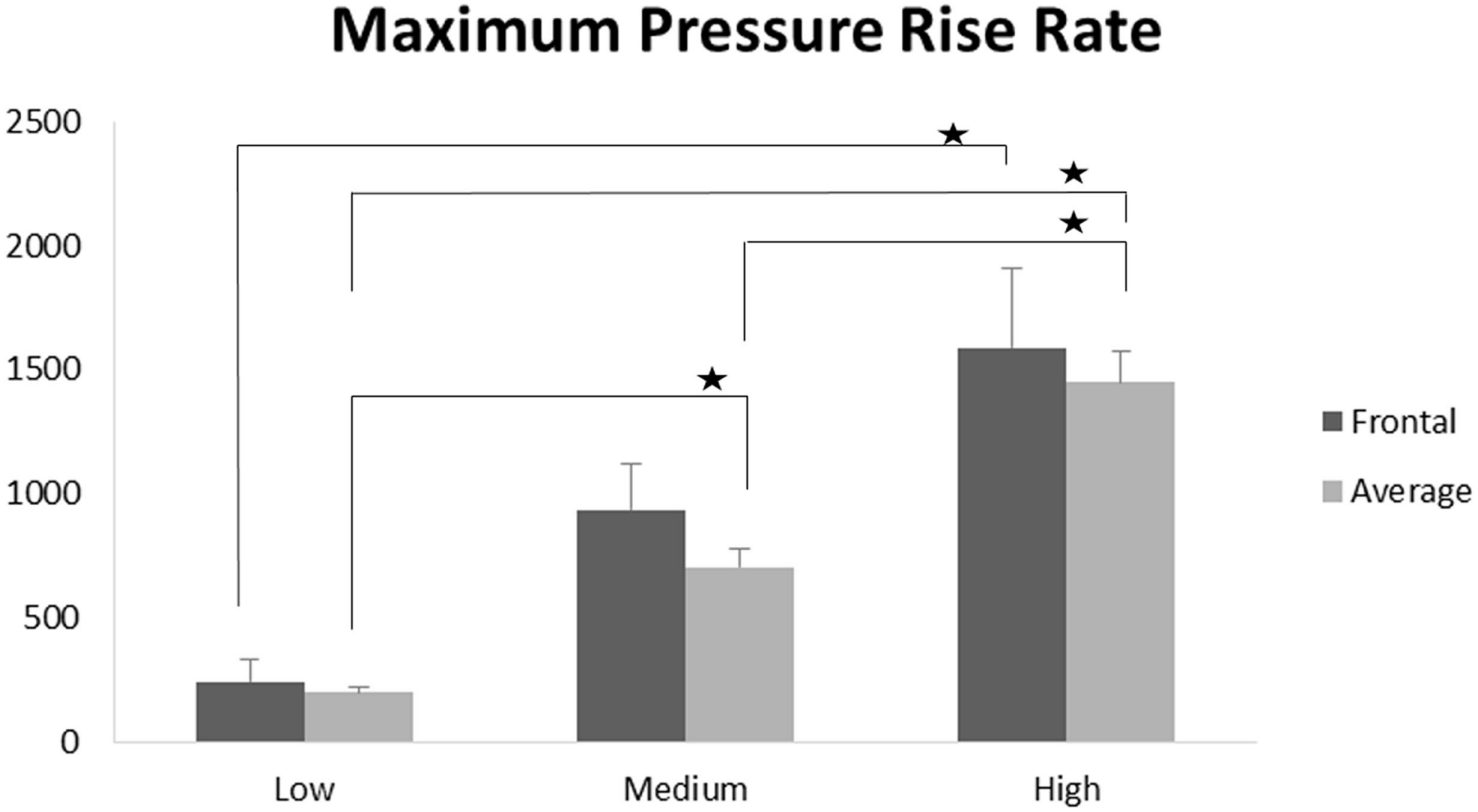
Maximum pressure rise rates were significantly increased with increasing blast levels both in the frontal region and when averaged across all five locations (*p < 0.05).

**Figure 8:**
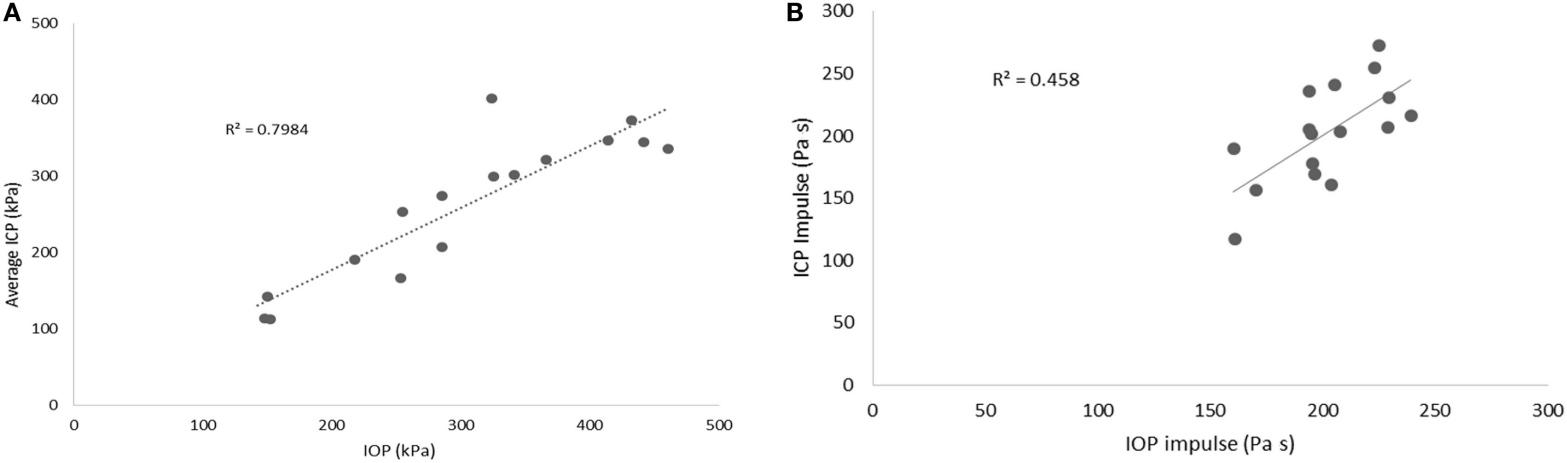
**(A)** Scatter plots of average ICP peak values at different locations vs. IOP. **(B)** Average ICP impulse at different locations vs. IOP impulse.

Scatter plots show that peak ICPs increased with peak IOP at every instrumented location (Figure 4). More specifically, ICP peak values correlated well with peak IOP in all the three blast pressure levels using linear regression models. The overall ICP responses were close or lower than its IOP at each blast level. This result is not unreasonable because the sensors were not at the brain/skull junction where the ICP is expected to be higher due to impedance mismatch. At the low blast level, peak ICP responses in the occipital and center regions were significantly lower than the peak IOPs (paired *t*-test, *p* < 0.05), with no significant differences in other regions of the brain (paired *t*-test, *p* > 0.05). At the medium blast level, no significant difference was found between peak ICP responses and peak IOP (paired *t*-test, *p* > 0.05). At the high blast level, peak ICPs were not significantly different from the peak IOPs (paired *t*-test, *p* > 0.05), except that in the center regions where the peak ICPs were significantly lower compared with the peak IOPs (paired *t*-test, *p* < 0.05) (Figure 5). This means that ICP drops as it traverses the brain but the drop is not significant. There was no statistically significance difference in peak ICPs between various locations at the low, medium, and high levels (ANOVA, *p* > 0.05). Statistical analysis also showed significant differences in the peak ICP between the medium and the high blast levels (ANOVA, *post hoc* LSD, *p* < 0.05) (Figure 6), indicating that change in the ICP are larger at higher IOP levels. The average maximum pressure rise rate increased significantly with blast levels. A similar trend was observed in the frontal region where the maximum pressure rise rates were higher (Figure 7). That is, the pressure rise rate is a function of the magnitude of the pressure and this observation should be relatable to the observed injuries in the brain. Average peak ICP peak values in each test correlated well with peak IOPs (Figure 8A). Average ICP impulses of each test were also correlated with IOP impulses (Figure 8B).

### Head Kinematics

**Figure 9:**
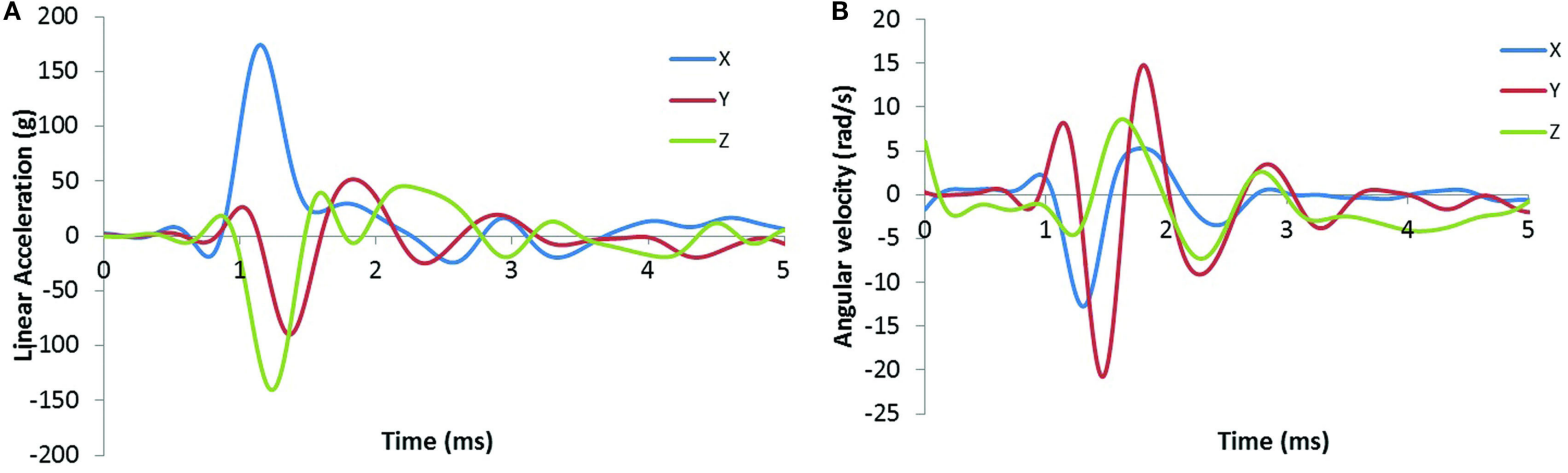
**Sample time-history plots of the acceleration and angular rate measured on the swine head with instrumentation mounted to the skull**. **(A)** The left plot shows linear acceleration (g) in the *x, y*, and *z* directions. **(B)** The right plot shows angular velocity (rad/s) in the *x, y*, and *z* directions.

**Figure 10:**
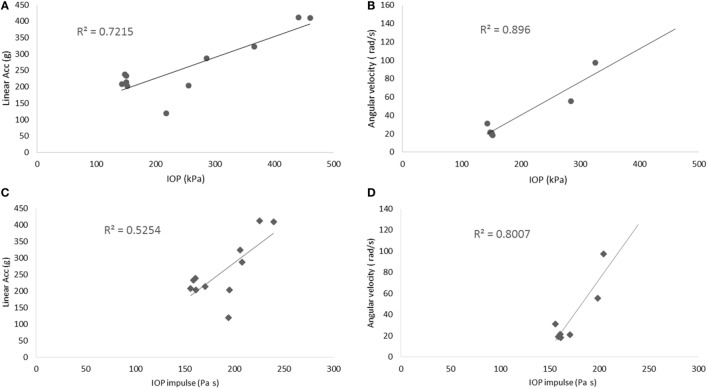
**Scatter plots of the motion of the head**. **(A,B)** show the relationship between the peak IOP (abscissa) and the resultant linear acceleration (ordinate, left graph), or the resultant angular velocity (ordinate, right graph). Similarly, **(C,D)** show the relationship between the IOP impulse (abscissa) and the resultant linear acceleration (ordinate, left graph), or the resultant angular velocity (ordinate, right graph). These variables correlated well with the peak IOP in linear regression models.

In this study, we characterized the head motion with its linear acceleration and angular velocity. Typical histories of the three linear accelerometers and the three angular rate sensors are shown in Figures 9A,B, respectively. The resultant linear accelerations and the resultant angular velocities increased linearly with peak IOP (Figures 10A,B). They were also well correlated with IOP impulses (Figures 10C,D). The resultant accelerations at high blast levels were significantly higher than those at the low and medium blast levels (ANOVA, *post hoc* LSD, *p* < 0.05), but there was no statistical significance between the low and the medium blast levels (ANOVA, *post hoc* LSD, *p* > 0.05). The resultant angular velocity at the medium blast level was significantly higher than that at the low blast level (independent *t-test, p* < 0.05). Here again, there is a non-linear relationship between IOP and head response that increases in severity as the IOP increases. This is a factor that needs to be considered in the design of protective gear. It may not be possible to protect those in extreme environments. The durations of the linear acceleration were typically less than 3 ms, indicating that there was little translational movement of the head during primary blast.

## Discussion

Animals are commonly used to study TBI (2, 31). In this study, we chose the Yucatan pig as their body mass and skull thickness are closer to those of the human than small animals.

This study exposed live swine subjects to free-field blast loading at various pressures and durations by changing the standoff distance between the charge and the swine. Of the five swine tested, two expired just before the blast testing, and one died during the tests. This resulted in 8 of the 19 blasts being performed on expired animals. However, the ICP responses showed little difference between expired animals and live animals. The potential causes of death could be related to complications from anesthesia and surgical procedure to insert ICP sensors. Additionally, a parallel group of non-instrumented animals were subjected to a single blast exposure (range 222–403 kPa). Observation of the brain harvested after perfusion performed after 3 days of survival showed no gross injury. Initial histological results from the frontal sections of the blast showed evidence of neuronal injury in the form of beta amyloid precursor protein immunoreactive zones in the gray and white matter. Neuronal injury was also supported by neurofilament light chain immunohistochemistry. Furthermore, an obvious increase in the number of astrocytes and microglia was also observed in the blast-exposed sections compared to sham sections. We hypothesize that there is direct correlation between ICP and brain injuries. Our histological studies will be testing this hypothesis.

In this study, we analyzed the ICP in different regions of the brain at various blast IOP levels. All previous studies have addressed the mechanical responses of the brain to blast with post-mortem human subjects (PMHS) (32), rats (20, 22, 33–35), and swine (19, 23, 36, 37) models using compressed-gas shock tubes in a laboratory environment or blast tube. Although some of these models provided crucial information on the correlation between IOP levels and injury responses, challenges with shock tube tests still exist, including animal positioning, orientation, and interpretation of the effect of the relatively longer duration of the blast (38). One previous animal model placed the animal head right outside of shock tube (23). It brought dramatic changes to the IOP characteristics, including the formation of a strong vortex flow and elevated dynamic blast pressure and impulse, which can have a combination of primary/tertiary effects quite different from our free-field tests. Data from rat blast models tested in shock tubes recorded positive phase durations in the range of 4–18 ms (20, 22, 33, 35), which is longer than blasts in the real world. Without sound scaling laws developed between species of bTBI models, shock tube test results need to be carefully investigated and compared with free-field explosive detonations. In this study, all experiments were performed in an open field blast environment. To minimize multiple waveforms from ground reflections, we placed the animal below the triple point and exposed it to the Mach stem. The IOPs were typical free-field Friedlander blast waves in the Mach stem region based on our analysis of the IOP data.

This study provided detailed ICP response in the swine brain subjected to free-field blasts. Historically, some animal tests have been designed and carried out in an attempt to investigate the mechanism of shock wave transmission to the brain, but only a few animal studies recorded direct pressure within the brain tissue during exposure to blast (20, 22, 23, 37). In our study, the results have demonstrated that ICP followed a trend of increasing magnitude with increased blast severity.

The relationship between ICP and IOP has been determined in several animal blast studies. We showed that, at different locations in the brain, peak ICP values were close to or lower than the IOP. One similar observation was made by another group investigating the mechanical response of the swine brain subjected to left-sided blasts in a shock tube (23). The peak IOPs ranged from 110 to 740 kPa with scaled durations from 1.3 to 6.9 ms. ICPs ranged from 80 to 390 kPa and were lower than the IOPs and notably lower than the reflected pressures of 300–2830 kPa. Another swine study by Bauman was performed in both a blast tube and in a simulated building with frontal blasts (19). The recorded IOP data showed that the test animal was exposed to multiple shock waveforms. Fiber optic pressure transducers were used to record pressure from within the forebrain, thalamus, and hindbrain of the swine without specifying details related to the locations of transducers. The ICP results showed that for IOP levels of 100–250 kPa, the peak ICP values at the three locations were lower than the IOPs (37).

In addition to swine, smaller animals, such as rats, have also been used. In a rat study, an optic fiber pressure sensor was used to record shock tube-generated ICPs. The animals were exposed to a low-level blasts of about 40 kPa and the recorded peak ICPs were close to but lower than the IOP in both the frontal and lateral regions of the brain (20). However, this study only used one ICP sensor in each test, and the results of the study were not statistically analyzed. There were also some discrepancies between findings in the peak ICP values compared to the peak IOP values in rat models. Leonardi et al. reported that peak ICPs in rats were larger than the peak IOPs and suggested that skull flexure due to an immature skull suture could be the source of the pressure increase (22). One recent study with cadaver rats also showed a higher peak ICP compared to the peak IOP values at different IOP levels (39). However, the location of the ICP sensor in the brain was not described, and the torso was not properly shielded from the shockwave. Also, the impulse produced in this study was in the range of 165–497 Pa s, larger than what we used in this study (160–240 Pa s).

Blast studies have also been performed on PMHS. In one PMHS study, using a shock tube, four fiber optic sensors were implanted in the right frontal cortex, right lateral ventricle, right parietal lobe, and right occipital lobe with the respective depths of the tip of the sensors from the outer surface of the skull being 30, 30, 65, and 30 mm (32). At each IOP level, the peak ICP values in the frontal lobe were higher than its peak IOP value. This observation was not seen at other locations of the brain. Also, most of the computer models indicated higher peak ICP compared with IOP readings (13, 27, 37, 40–42).

The discrepancy between measured and model predicted ICP and IOP readings could be due to several causes. One would be the highly non-linear relationship between the ICP at various locations and the IOP wave (37). Due to the impedance mismatch between the skull and the brain, ICP peak values tend to be higher at the boundaries and lower in the central region (43). With respect to the location of transducers, computer models can precisely pinpoint the coup and countercoup regions of the brain. The location of the ICP sensors in animal experiments was limited by surgical techniques. The depths of sensors below the skull in all experimental tests were different or not described in detail. Therefore, the ICP readings varied in the published literature as described above. Another reason for ICP differences seen in rats and pig is possibly due to the morphological differences between species. Compared to rats, pigs have a much thicker skull with a complex dipole layer that is full of voids. Computer models, on the other hand, may have oversimplified the skull and yielded predictions that did not match experimental data.

**Figure 11:**
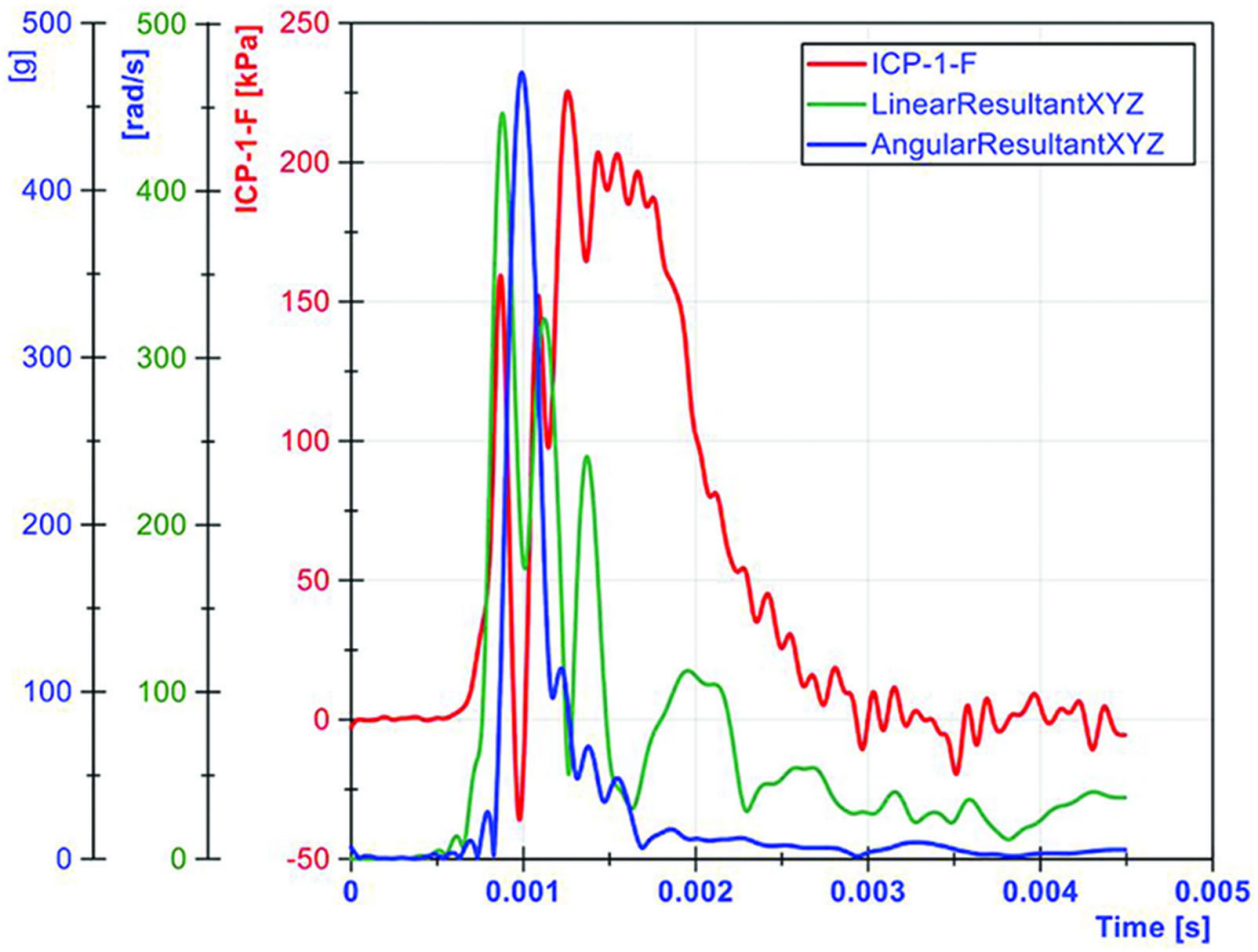
**The relationship between ICP and head motion (resultant linear head acceleration and angular velocity) demonstrated that primary blast imparted a severe acceleration to the head, albeit the duration was very short**.

**Figure 12:**
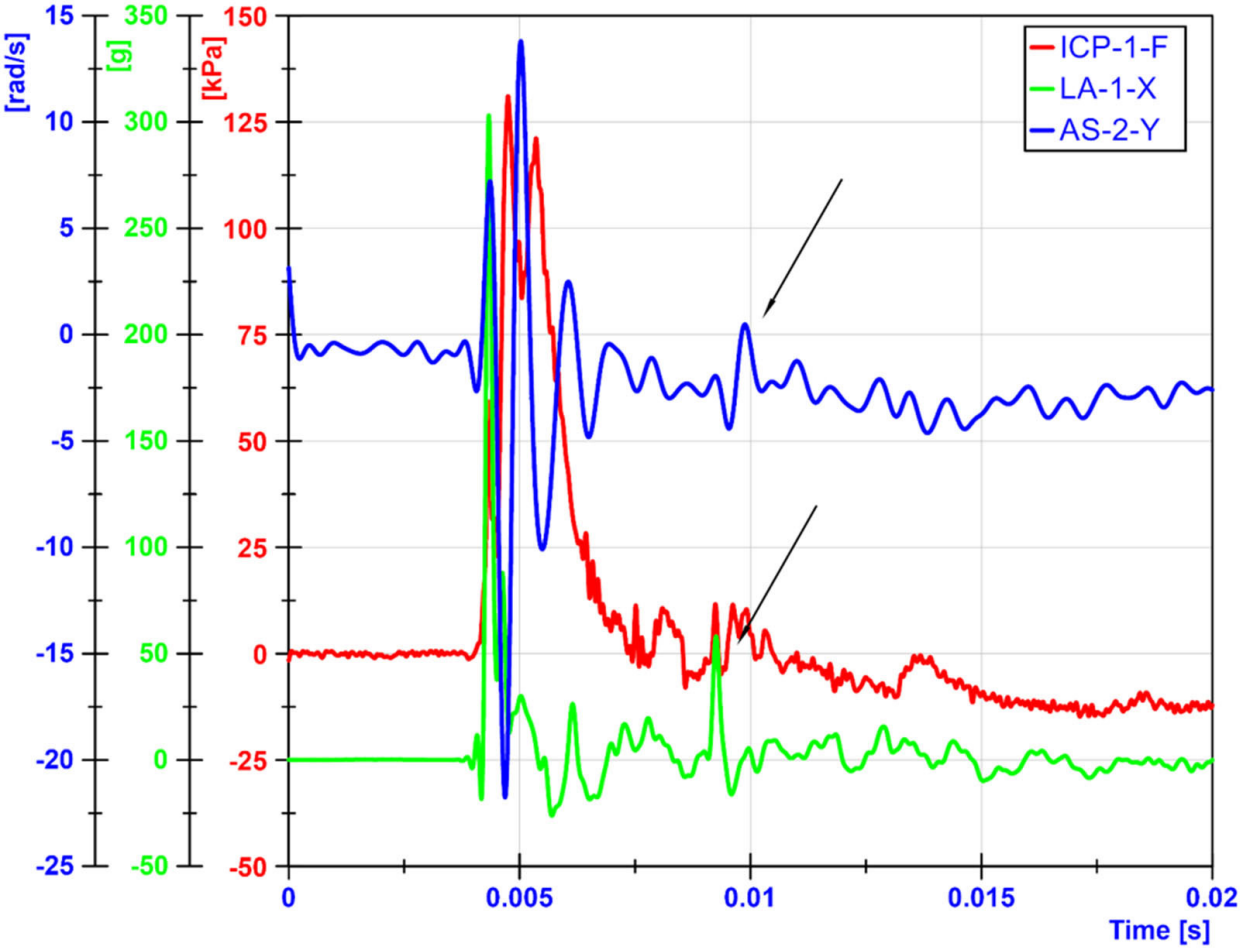
**Head motions due blast winds occurred around 5 ms later than motions from primary shockwaves**. The arrows show when the blast wind induced head movements.

Both linear and angular motions of the head were acquired in our tests. The arrival of the ICP wave was almost simultaneous with head motion. Thus, the head motion was due to the primary blast wave (Figure 11). However, the duration of the motion was relatively short (1–2 ms), which resulted in the maximum head displacement around 2 mm. Similar observations were made by Shridharani et al. who also used swine subjects (23). They found strong correlations (*R*^2^ = 0.9) between peak resultant acceleration and peak IOP in the range of 110–740 kPa in a linear model. Their positive phase duration was around 3 ms, and the maximum head displacement was 7.5 mm. Thus, the observed acceleration in these two studies was likely due to the primary shock wave. Well after the passage of the shock wave, we observed inertial global head movement but the head acceleration due to the blast wind was not significant compared to the initial acceleration due to the primary shock wave (Figure 12). In this study, we have deliberately avoided using HIC as an injury measure because HIC was developed for blunt impact with much longer durations and its validity for acceleration pulses lasting only a few milliseconds is questionable.

The data reported here were acquired from live, anesthetized swine exposed to primary blast waves. This is the first large animal model exposed to free-field blasts in which detailed internal pressure measurements were made at various locations. Head motion due to primary blast waves was also measured. The mechanical responses of swine need to be scaled to the human head to determine human response. However, due to the morphological differences between the two species, scaling laws can be difficult to develop. The direct translation can only be done by finite element modeling to develop tissue level response correlates for swine brain. This tissue level response threshold can be then directly translated to the human brain model enabling the development of blast injury threshold for human. Also, the limited sample size should also be taken into consideration. Due to time limitations to complete nine blast tests on a single animal in 8 h and failure of the data acquisition system on one occasion, data were available from only 19 tests on 5 animals. Additional testing of more animals should improve the statistical significance of the results.

## Conclusion

In summary, the results of this study provided a set of detailed biomechanical response data of swine skull and brain during exposure to primary blast waves, with the peak IOPs ranging from 143 to 461 kPa, and the impulses ranging from 156 to 239 Pa s. The overall ICP responses were closer to or lower than its IOP at each blast level. More specifically, peak ICP values at the frontal, parietal, and temporal were statistically the same as the corresponding IOP values. Peak ICP values at the frontal, central, and occipital regions were significantly elevated at the medium and high blast levels compared with the low blast levels. Furthermore, only at the central location, was the ICP significantly different between the medium and high pressures tests. Both the linear acceleration and the angular velocity increased with blast levels. Although the head acceleration was high, its duration was less than 2 ms. It is unlikely that the brain would be able to respond mechanically to this type of acceleration input. The experimental data can be used to validate computer models.

## Author Contributions

AK, JC, and LZ designed the experiment; KF, XJ, CC, TS, SK, and LZ performed the experiment; and KF, JC, and AK drafted the manuscript.

## Conflict of Interest Statement

The authors declare that the research was conducted in the absence of any commercial or financial relationships that could be construed as a potential conflict of interest.

## References

[1] Mac Donald CL Johnson AM Cooper D Nelson EC Werner NJ Shimony JS Detection of blast-related traumatic brain injury in U.S. military personnel. N Engl J Med (2011) 364:2091–100. 10.1056/NEJMoa1008069 21631321 PMC3146351

[2] Risling M Davidsson J. Experimental animal models for studies on the mechanisms of blast-induced neurotrauma. Front Neurol (2012) 3:30. 10.3389/fneur.2012.00030 22485104 PMC3317041

[3] Nakagawa A Manley GT Gean AD Ohtani K Armonda R Tsukamoto A Mechanisms of primary blast-induced traumatic brain injury: insights from shock-wave research. J Neurotrauma (2011) 28:1101–19. 10.1089/neu.2010.1442 21332411

[4] Bass CR Panzer MB Rafaels KA Wood G Shridharani J Capehart B. Brain injuries from blast. Ann Biomed Eng (2012) 40:185–202. 10.1007/s10439-011-0424-0 22012085

[5] Richmond DR Damon EG Bowen IG Fletcher ER White CS Air-Blast Studies with Eight Species of Mammals. Fission Product Inhalation Project [Technical Progress Report]. (1967). Techn Progr Rep DASA 1854. Lovelace Foundation for Medical Education and Research. 1–44. 5302772

[6] Richmond DR Damon EG Fletcher ER Bowen IG White CS The Relationship between Selected Blast-Wave Parameters and the Response of Mammals Exposed to Air Blast. Fission Product Inhalation Project [Technical Progress Report]. (1967). Techn Progr Rep DASA 1860. Lovelace Foundation for Medical Education and Research. 1–36. 4970917

[7] White CS Bowen IG Richmond DR Biological Tolerance to Air Blast and Related Biomedical Criteria. Civil Effects Exercise. (1965). CEX-65.4. CEX [Reports]. U.S. Atomic Energy Commission. 1–239. 10.2172/4614491 5853741

[8] Wood GW Panzer MB Shridharani JK Matthews KA Capehart BP Myers BS Attenuation of blast pressure behind ballistic protective vests. Inj Prev (2013) 19:19–25. 10.1136/injuryprev-2011-040277 22544830

[9] Kennedy JE Jaffee MS Leskin GA Stokes JW Leal FO Fitzpatrick PJ. Posttraumatic stress disorder and posttraumatic stress disorder-like symptoms and mild traumatic brain injury. J Rehabil Res Dev (2007) 44:895–920. 10.1682/JRRD.2006.12.0166 18075948

[10] Heltemes KJ Holbrook TL Macgregor AJ Galarneau MR. Blast-related mild traumatic brain injury is associated with a decline in self-rated health amongst US military personnel. Injury (2012) 43:1990–5. 10.1016/j.injury.2011.07.021 21855064

[11] Elder GA Cristian A. Blast-related mild traumatic brain injury: mechanisms of injury and impact on clinical care. Mt Sinai J Med (2009) 76:111–8. 10.1002/msj.20098 19306373

[12] Gupta RK Przekwas A. Mathematical models of blast-induced TBI: current status, challenges, and prospects. Front Neurol (2013) 4:59. 10.3389/fneur.2013.00059 23755039 PMC3667273

[13] Zhang L Makwana R Sharma S. Brain response to primary blast wave using validated finite element models of human head and advanced combat helmet. Front Neurol (2013) 4:88. 10.3389/fneur.2013.00088 23935591 PMC3731672

[14] Przekwas A Tan X Harrand V Reeves D Chen Z Sedberry K Integrated experimental and computational framework for the development and validation of blast wave brain biomechanics and helmet protection. Proc. HFM-207 NATO Symposium on a Survey of Blast Injury Across the Full Landscape of Military Science. (2011).

[15] Rafaels KA Bass CR Panzer MB Salzar RS Woods WA Feldman SH Brain injury risk from primary blast. J Trauma Acute Care Surg (2012) 73:895–901. 10.1097/TA.0b013e31825a760e 22836001

[16] Reneer DV Hisel RD Hoffman JM Kryscio RJ Lusk BT Geddes JW. A multi-mode shock tube for investigation of blast-induced traumatic brain injury. J Neurotrauma (2011) 28:95–104. 10.1089/neu.2010.1513 21083431 PMC3019584

[17] Sundaramurthy A Chandra N. A parametric approach to shape field-relevant blast wave profiles in compressed-gas-driven shock tube. Front Neurol (2014) 5:253. 10.3389/fneur.2014.00253 25520701 PMC4251450

[18] Ben-Dor G Shock Wave Reflection Phenomena. Springer (2007).

[19] Bauman RA Ling G Tong L Januszkiewicz A Agoston D Delanerolle N An introductory characterization of a combat-casualty-care relevant swine model of closed head injury resulting from exposure to explosive blast. J Neurotrauma (2009) 26:841–60. 10.1089/neu.2009-0898 19215189

[20] Chavko M Koller WA Prusaczyk WK McCarron RM. Measurement of blast wave by a miniature fiber optic pressure transducer in the rat brain. J Neurosci Methods (2007) 159:277–81. 10.1016/j.jneumeth.2006.07.018 16949675

[21] Long JB Bentley TL Wessner KA Cerone C Sweeney S Bauman RA. Blast overpressure in rats: recreating a battlefield injury in the laboratory. J Neurotrauma (2009) 26:827–40. 10.1089/neu.2008.0748 19397422

[22] Leonardi AD Bir CA Ritzel DV VandeVord PJ. Intracranial pressure increases during exposure to a shock wave. J Neurotrauma (2011) 28:85–94. 10.1089/neu.2010.1324 21091267

[23] Shridharani JK Wood GW Panzer MB Capehart BP Nyein MK Radovitzky RA Porcine head response to blast. Front Neurol (2012) 3:70 10.3389/fneur.2012.00070 22586417 PMC3347090

[24] Nyein MK Jason AM Yu L Pita CM Joannopoulos JD Moore DF In silico investigation of intracranial blast mitigation with relevance to military traumatic brain injury. Proc Natl Acad Sci U S A (2010) 107:20703–8. 10.1073/pnas.1014786107 21098257 PMC2996433

[25] Moss WC King MJ Blackman EG. Skull flexure from blast waves: a mechanism for brain injury with implications for helmet design. Phys Rev Lett (2009) 103:108702. 10.1103/PhysRevLett.103.108702 19792349

[26] Lockhart P Cronin D Williams K Ouellet S. Investigation of head response to blast loading. J Trauma (2011) 70:E29–36. 10.1097/TA.0b013e3181de3f4b 20664376

[27] Chafi MS Karami G Ziejewski M. Biomechanical assessment of brain dynamic responses due to blast pressure waves. Ann Biomed Eng (2010) 38:490–504. 10.1007/s10439-009-9813-z 19806456

[28] Wang C Pahk JB Balaban CD Miller MC Wood AR Vipperman JS. Computational study of human head response to primary blast waves of five levels from three directions. PLoS One (2014) 9:e113264. 10.1371/journal.pone.0113264 25409326 PMC4237386

[29] De Rosa M Fam F Palleschi VV Singh DP Vaselli M Derivation of the critical angle for Mach reflection for strong shock waves. Phys Rev A (1992) 45:6130–2. 10.1103/PhysRevA.45.6130 9907718

[30] Ivanov MS Vandromme D Fomin VM Kudryavtsev AN Hadjadj A Khotyanovsky DV Transition between regular and Mach reflection of shock waves: new numerical and experimental results. Shock Waves (2001) 11:199–207. 10.1007/PL00004075

[31] Cernak I Blast-induced neurotrauma models and their requirements. Front Neurol (2014) 5:128 10.3389/fneur.2014.00128 25071713 PMC4091031

[32] Bir C Measuring Blast-Related Intracranial Pressure within the Human Head. DTIC Document (2011).

[33] Huber BR Meabon JS Martin TJ Mourad PD Bennett R Kraemer BC Blast exposure causes early and persistent aberrant phospho- and cleaved-tau expression in a murine model of mild blast-induced traumatic brain injury. J Alzheimers Dis (2013) 37:309–23. 10.3233/JAD-130182 23948882 PMC4126588

[34] Gullotti DM Beamer M Panzer MB Chen YC Patel TP Yu A Significant head accelerations can influence immediate neurological impairments in a murine model of blast-induced traumatic brain injury. J Biomech Eng (2014) 136:091004. 10.1115/1.4027873 24950710

[35] Goldstein LE Fisher AM Tagge CA Zhang XL Velisek L Sullivan JA Chronic traumatic encephalopathy in blast-exposed military veterans and a blast neurotrauma mouse model. Sci Transl Med (2012) 4:134ra60 10.1126/scitranslmed.3004862 PMC3739428 22593173

[36] Saljo A Arrhen F Bolouri H Mayorga M Hamberger A. Neuropathology and pressure in the pig brain resulting from low-impulse noise exposure. J Neurotrauma (2008) 25:1397–406. 10.1089/neu.2008.0602 19146459

[37] Zhu F Skelton P Chou CC Mao H Yang KH King AI Biomechanical responses of a pig head under blast loading: a computational simulation. Int J Numer Method Biomed Eng (2013) 29:392–407. 10.1002/cnm.2518 23345257

[38] Needham CE Ritzel D Rule GT Wiri S Young L. Blast testing issues and TBI: experimental models that lead to wrong conclusions. Front Neurol (2015) 6:72. 10.3389/fneur.2015.00072 25904891 PMC4389725

[39] Skotak M Wang F Alai A Holmberg A Harris S Switzer RC Rat injury model under controlled field-relevant primary blast conditions: acute response to a wide range of peak overpressures. J Neurotrauma (2013) 30:1147–60. 10.1089/neu.2012.2652 23362798 PMC3700437

[40] Moore DF Jerusalem A Nyein M Noels L Jaffee MS Radovitzky RA. Computational biology – modeling of primary blast effects on the central nervous system. Neuroimage (2009) 47(Suppl 2):T10–20. 10.1016/j.neuroimage.2009.02.019 19248833

[41] Zhu F Mao H Dal Cengio Leonardi A Wagner C Chou C Jin X Development of an FE model of the rat head subjected to air shock loading. Stapp Car Crash J (2010) 54:211–25. 21512910 10.4271/2010-22-0011

[42] Taylor PA Ford CC. Simulation of blast-induced early-time intracranial wave physics leading to traumatic brain injury. J Biomech Eng (2009) 131:061007. 10.1115/1.3118765 19449961

[43] Meyers MA Dynamic Behavior of Materials. New York: Wiley (1994).

